# Alterations in brain neurocircuitry following treatment with the chemotherapeutic agent paclitaxel in rats

**DOI:** 10.1016/j.ynpai.2019.100034

**Published:** 2019-05-27

**Authors:** Craig F. Ferris, Sarah Nodine, Trent Pottala, Xuezhu Cai, Tatiana M. Knox, Fanta H. Fofana, Soojin Kim, Praveen Kulkarni, Jonathon D. Crystal, Andrea G. Hohmann

**Affiliations:** a Center for Translational NeuroImaging, Northeastern University, Boston, MA, United States; b Depts Psychology and Pharmaceutical Sciences, Northeastern University, United States; c Dept of Psychology and Brain Sciences, Indiana University, Bloomington, IN, United States

## Abstract

Human and animal studies suggest that both traumatic nerve injury and toxic challenge with chemotherapeutic agents involves the reorganization of neural circuits in the brain. However, there have been no prospective studies, human or animal, using magnetic resonance imaging (MRI) to identify changes in brain neural circuitry that accompany the development of chemotherapy-induced neuropathic pain (i.e. within days following cessation of chemotherapy treatment and without the confound cancer). To this end, different MRI protocols were used to ascertain whether a reorganization of brain neural circuits is observed in otherwise normal rats exposed to the taxane chemotherapeutic agent paclitaxel.

We conducted an imaging study to evaluate the impact of a well-established paclitaxel dosing regimen, validated to induce allodynia in control rats within eight days of treatment, on brain neural circuitry. Rats received either paclitaxel (2 mg/kg/day i.p; cumulative dose of 8 mg/kg) or its vehicle four times on alternate days (i.e. day 0, 2, 4, 6). Following the cessation of treatments (i.e. on day 8), all rats were tested for responsiveness to cold followed by diffusion weighted magnetic resonance imaging and assessment of resting state functional connectivity. Imaging data were analyzed using a 3D MRI rat with 173 segmented and annotated brain areas.

Paclitaxel-treated rats were more sensitive to a cold stimulus compared to controls. Diffusion weighted imaging identified brain areas involved in the emotional and motivational response to chronic pain that were impacted by paclitaxel treatment. Affected brain regions included the prefrontal cortex, amygdala, hippocampus, hypothalamus and the striatum/nucleus accumbens. This putative reorganization of gray matter microarchitecture formed a continuum of brain areas stretching from the basal medial/lateral forebrain to the midbrain. Resting state functional connectivity showed reorganization between the periaqueductal gray, a key node in nociceptive neural circuitry, and connections to the brainstem.

Our results, employing different imaging modalities to assess the central nervous system effects of chemotherapy, fit the theory that chronic pain is regulated by emotion and motivation and influences activity in the periaqueductal gray and brainstem to modulate pain perception.

## Highlights

• Imaging the reorganization of pain neural circuitry within 8 days of chemotherapy.
• Using rat model of neuropathy with multimodal MRI.
• Showing loss of anticorrelation between prefrontal cortex and PAG.
• Identifying the interaction between periaqueductal gray and brainstem raphe.

## 1 Introduction

Cancer statistics are equally puzzling and humbling—in 2018, over 1.7 million new cancer cases and 600,000 cancer-related deaths are projected to occur in the United States alone (Siegel et al., 2018). Though grim, those statistics do not speak to the concurrent 76% drop in combined US cancer deaths since the early 1990s. The benefits of improving cancer treatments and decreasing mortality rates have given rise to a global survivor population of over 32 million. Chemotherapy is an indispensable treatment option for many common cancers and is used in neoadjuvant, adjuvant, and metastatic settings. Unfortunately, chemotherapy-induced peripheral neuropathy (CIPN) affects as many as half of singly-treated patients and more than 75% of those treated with combination chemotherapies (Zhang et al., 2016). CIPN is characterized by chronic shooting or burning pain and loss of sensation that begins at the extremities and progresses more centrally; many also report mechanical sensitivity and thermal allodynia/hyperalgesia (Fukuda et al., 2017).

Paclitaxel, is a commonly used chemotherapeutic for the treatment of breast, lung, ovarian, and pancreatic cancer (Gangloff et al., 2005). Paclitaxel arrests mitosis through its interactions with β-tubulin, a component of microtubules (Flatters and Bennett, 2006). Most cells, but especially those which divide more often, are susceptible to these anti-mitotic effects. Though neurons are not explicitly “dividing” cells, peripheral sensory axons are nonetheless susceptible to similar paclitaxel-induced degeneration. Peripheral sensory axons in paclitaxel models are marked by the formation of retraction bulbs, a biomarker of degeneration that likely accounts for the particular occurrence of sensory peripheral neuropathies, since sensory neurons rely on pronounced distal growth to constantly reinnervate regenerating epidermis. In rats, paclitaxel accumulates in dorsal root ganglion (DRG) cells, with limited penetration distal to the DRG in the sciatic nerve and proximal to the DRG in the spinal cord, with the lowest concentrations measured in the brain (Cavaletti et al., 2000). Thus, most preclinical studies evaluating mechanisms underlying development and maintenance of paclitaxel-induced neuropathic pain have focused on dorsal root ganglia, primary afferents and their sites of termination in the spinal cord and periphery. Comprehensive studies assessing the distribution, magnitude and extent of paclitaxel-induced changes in brain neurocircuitry in rodents are, consequently, needed.

There have been numerous imaging studies in humans characterizing changes in corticothalamic circuitry associated with cold allodynia following injury to the spinothalamic tracts (for review see (Peyron, 2016). Human imaging studies on chronic pain in general report changes in gray matter and functional connectivity between a network of core brain areas e.g., prefrontal cortex, accumbens, basal ganglia, thalamus, anterior cingulate and periaqueductal gray, emphasizing the influence of emotion and motivation on the perception of chronic pain (Cauda et al., 2014, Baliki et al., 2010, Baliki et al., 2011, Baliki et al., 2012, Mills et al., 2018) There have been no imaging studies in animals on chemotherapy induced neuropathy. However, there have been several imaging studies in rodents using the spared nerve injury (SNI) model (Chang et al., 2014, Baliki et al., 2014, Hubbard et al., 2015, Bilbao et al., 2018, Seminowicz et al., 2009) to create neuropathy. Findings from these studies corroborate the human data and report a reorganization around the prefrontal ctx, basal ganglia, accumbens and periaqueductal gray. These translational results led us to hypothesize that paclitaxel treatment would reorganize pain neural circuitry around brain areas involved in emotion and motivation. The present study in rats uses diffusion weighted imaging with quantitative anisotropy to follow changes in gray matter microarchitecture and resting state functional connectivity to assess brain reorganization following paclitaxel treatment. The findings fit the theory that chronic pain is regulated by emotion and motivation that influence activity in the periaqueductal grey and brainstem to modulate pain perception.

## 2 Methods

### 2.1 Animals

Male Sprague Dawley rats (n = 16) weighing between 300 and 325 gm were obtained from Charles River Laboratories (Wilmington, Massachusetts, USA). Rats were maintained on a 12:12 h light:dark cycle with a lights on at 07:00 h and allowed access to food and water ad libitum. All rats were acquired and cared for in accordance with the guidelines published in the Guide for the Care and Use of Laboratory Animals (National Institutes of Health Publications No. 85–23, Revised 1985) and adhered to the National Institutes of Health and the American Association for Laboratory Animal Science guidelines. The protocols used in this study complied with the regulations of the Institutional Animal Care and Use Committee at the Northeastern University.

### 2.2 Drugs

Paclitaxel was purchased from Tecoland Corporation (Irvine, CA). Paclitaxel was dissolved in a vehicle consisting of cremophor EL: ethanol: saline to achieve a final concentration of 1:1:18. Rats received either paclitaxel (2 mg/kg/day i.p.; cumulative dose of 8 mg/kg i.p.) or its cremophor-based vehicle four times on alternate days (i.e. day 0, 2, 4, 6) using methods validated in our laboratories to produce mechanical allodynia, cold allodynia, reversal learning deficits and reductions in hippocampal neurogenesis in rats (Carey et al., 2017, Deng et al., 2012, Deng et al., 2016, Lee et al., 2015, Panoz-Brown et al., 2017, Rahn et al., 2008, Rahn et al., 2014, Smith et al., 2017). We previously reported that paclitaxel-induced allodynia developed by day 7 and was maintained for at least 3 months following initiation of paclitaxel dosing (Deng et al., 2016, Panoz-Brown et al., 2017, Rahn et al., 2014, Smith et al., 2017). Paclitaxel and cremophor-based vehicle were prepared in the lab of Dr. Hohmann at Indiana University and sent to the CTNI coded as treatment A and B. The staff at the CTNI were blind to the treatments. It was only after all of the behavior and imaging were completed and analyzed was the code revealed to all investigators.

### 2.3 Cold plate assay

The cold plate assay to test for cold allodynia was taken from Brenner and Golden (2012) and modified for rats. Prior to testing, rats were acclimated to standing on a ¼ inch glass plate table, confined to an area of 38 cm × 14 cm by a transparent plastic container. Beneath the glass table a mirror was positioned to permit a clear view of the rat’s hind paws for the precise placement of the cold probe. A hollow probe with a tip dimension of 2 mm was filled with compressed, crushed dry ice. The center of the plantar surface of the hindpaw was targeted for stimulation through the floor of the glass platform. The cold probe was tested on each hindpaw with an interval of 7 min between each paw. Both hindpaws of each rat were tested three times with an interval of 15 min between trials. The latency to move the hindpaw away from the cold probe was timed in seconds. The maximum time allowed for withdrawal was 90 sec. Withdrawal latencies were averaged between the right and left hindpaw and did not differ significantly between paws within either the vehicle or paclitaxel treatments. Consequently, the right and left hindpaw withdrawal times were averaged for each animal, and a single mean determination from each animal contributed to the groups means and standard deviations for each treatment. These data were statistically compared with a Student’s t-Test. Experimenters collecting the data were blinded to the treatment condition. ‘

### 2.4 Neuroimaging

Imaging sessions were conducted using a Bruker Biospec 7.0 T/20-cm USR horizontal magnet (Bruker, Billerica, MA, USA) and a 20-G/cm magnetic field gradient insert (ID = 12 cm) capable of a 120-μs rise time. Radio frequency signals were sent and received with a quadrature volume coil built into the animal restrainer (Animal Imaging Research, Holden, Massachusetts). The design of the restraining system included a padded head support obviating the need for ear bars helping to reduce animal discomfort while minimizing motion artifact. All rats (n = 7 paclitaxel; n = 6 vehicle) were imaged under 1–2% isoflurane while keeping a respiratory rate of 40–50/min. At the beginning of each imaging session, a high-resolution anatomical data set was collected for volumetric analysis using the RARE pulse sequence with following parameters, 35 slice of 0.7 mm thickness; field of view [FOV] 3 cm; 256 × 256; repetition time [TR] 3900 msec; effective echo time [TE] 48 msec; NEX 3; 6 min 14 sec acquisition time.

### 2.5 Diffusion weighted imaging – quantitative anisotropy

DWI was acquired with a spin-echo echo-planar-imaging (EPI) pulse sequence having the following parameters: TR/TE = 500/20 ms, eight EPI segments, and 10 non-collinear gradient directions with a single b-value shell at 1000 s/mm^2^ and one image with a B-value of 0 s/mm^2^ (referred to as B_0_). Geometrical parameters were: 48 coronal slices, each 0.313 mm thick (brain volume) and with in-plane resolution of 0.313 × 0.313 mm^2^ (matrix size 96 × 96; FOV 30 mm^2^). The imaging protocol was repeated two times for signal averaging. Each DWI acquisition took 35 min and the entire MRI protocol lasted ca. 70 min. Image analysis included DWI analysis of the DW-3D-EPI images to produce the maps of fractional anisotropy (FA) and radial diffusivity (RD). DWI analysis was implemented with MATLAB and MedINRIA (1.9.0; http://www-sop.inria.fr/asclepios/software/MedINRIA/index.php) software. Because sporadic excessive breathing during DWI acquisition can lead to significant image motion artifacts that are apparent only in the slices sampled when motion occurred, each image (for each slice and each gradient direction) was screened, prior to DWI analysis, for motion artifacts; if found, acquisition points with motion artifacts were eliminated from analysis.

For statistical comparisons between rats, each brain volume was registered with the 3D rat atlas allowing voxel- and region-based statistics. All image transformations and statistical analyses were carried out using the in-house MIVA software. For each rat, the B_0_ image was co-registered with the B_0_ template (using a 6-parameter rigid-body transformation). The co-registration parameters were then applied on the DWI indexed maps for the different indices of anisotropy. Normalization was performed on the maps since they provided the most detailed visualization of brain structures and allow for more accurate normalization. The normalization parameters were then applied to all DWI indexed maps. The normalized indexed map was smoothed with a 0.3-mm Gaussian kernel. To ensure that the anisotropy values were not affected significantly by the pre-processing steps, the ‘nearest neighbor’ option was used following registration and normalization.

Statistical differences in measures of DWI between experimental groups were determined using a nonparametric Mann-Whitney U Test (alpha set at 5%). The formula below was used to account for false discovery from multiple comparisons.

$$P_{\left(i\right)}\leq \frac{i}{V}\frac{q}{c\left(V\right)}$$

*P(i)* is the *p* value based on the *t* test analysis. Each of 171 ROIs (i) within the brain containing (V) ROIs was ranked in order of its probability value (see Tables 1S-4S Supplementary Data). The false-positive filter value *q* was set to 0.2 and the predetermined *c(V)* was set to unity (Benjamini and Hochberg, 1995). The corrected probability is noted on each table.

### 2.6 Resting state functional connectivity

Resting-state fMRI was acquired with a gradient-echo triple-shot EPI sequence, TR/TE 3000/15 ms; matrix size 96 × 96 × 20; voxel size 0.312 × 0.312 × 0.12 mm; time points 200. Preprocessing in this study was accomplished by combining Analysis of Functional NeuroImages (AFNI_17.1.12, http://afni.nimh.nih.gov/afni/), FMRIB Software library (FSL, v5.0.9, http://fsl.fmrib.ox.ac.uk/fsl/), Deformable Registration via Attribute Matching and Mutual-Saliency Weighting (DRAMMS 1.4.1, https://www.cbica.upenn.edu/sbia/software/dramms/index.html) and MATLAB (Mathworks, Natick, MA). Brain tissue masks for resting-state functional images were manually drawn using 3DSlicer (https://www.slicer.org/) and applied for skull-stripping. Motion outliers (i.e., data corrupted by extensive motion) were detected in the dataset and the corresponding time points were recorded so that they could be regressed out in a later step. Functional data were assessed for the presence of motion spikes. Any large motion spikes were identified and removed from the time-course signals. This filtering step was followed by slice timing correction from interleaved slice acquisition order. Head motion correction (six motion parameters) was carried out using the first volume as a reference image. Normalization was completed by registering functional data to the 3D MRI Rat Brain Atlas© template (Ekam Solutions LLC, Boston, MA) using affine registration through DRAMMS. This pre-defined MRI rat brain atlas containing 171 brain regions was used for segmentation. Data are reported in 166 brain areas, as five regions in the brain atlas were excluded from analysis due to the large size of 3 brains. These brains fell slightly outside our imaging field of view and thus we did not get any signal from the extreme caudal tip of the cerebellum. Whole brains that contain all regions of interest are needed for analyses so rather than excluding the animals, we removed the brain sites across all animals. After quality assurance, band-pass filtering (0.01 Hz–0.1 Hz) was preformed to reduce low-frequency drift effects and high-frequency physiological noise for each subject. The resulting images were further detrended and spatially smoothed (full width at half maximum = 0.8 mm). Finally, regressors comprised of motion outliers, the six motion parameters, the mean white matter, and cerebrospinal fluid time series were fed into general linear models for nuisance regression to remove unwanted effects.

The region-to-region functional connectivity method was performed in this study to measure the correlations in spontaneous BOLD fluctuations. A network is comprised of nodes and edges; nodes being the brain region of interest (ROI) and edges being the connections between regions. 166 nodes were defined using the ROIs segmented from our custom MRI RAT Brain Atlas. Voxel time series data were averaged in each node based on the residual images using the nuisance regression procedure with motion parameters and mean time courses of white matter and ventricles. Pearson’s correlation coefficients across all pairs of nodes (13695 pairs) were computed for each subject among all three groups to assess the interregional temporal correlations. The r-values (ranging from −1 to 1) were z-transformed using the Fisher’s Z transform to improve normality. 166 × 166 symmetric connectivity matrices were constructed with each entry representing the strength of edge. Group-level analysis was performed to look at the functional connectivity in all experimental groups. The resulting Z-score matrices from one-group t-tests were clustered using the K-nearest neighbors clustering method to identify how nodes cluster together and form resting state networks. A Z-score threshold of |Z| = 2.3 was applied to remove spurious or weak node connections for visualization purposes

## 3 Results

### 3.1 Test for neuropathic pain

**Fig. 1:**
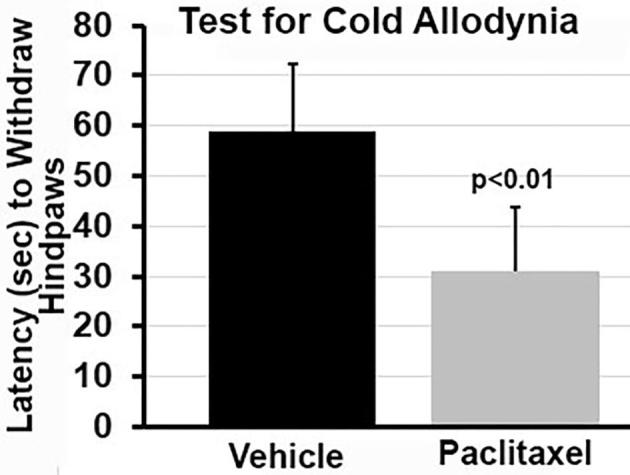
Cold Allodynia. The bar graphs show the mean (± SD) for the latency to withdraw the hindpaws in response to a cold stimulus. Rats (n = 9) treated with paclitaxel showed a significantly shorter latency (p < 0.001) than vehicle treated rats (n = 7).

The latency to withdraw the hindpaws in response to a cold stimulus probe was significantly lower (p < 0.001) in rats treated with paclitaxel, compared to rats receiving the cremophor-based vehicle (Fig. 1). Paclitaxel-treated rats exhibited approximately a 50% reduction in paw withdrawal latencies compared to their vehicle-treated counterparts, consistent with the development of robust cold allodynia.

### 3.2 Diffusion weighted imaging and quantitative anisotropy

**Fig. 2:**
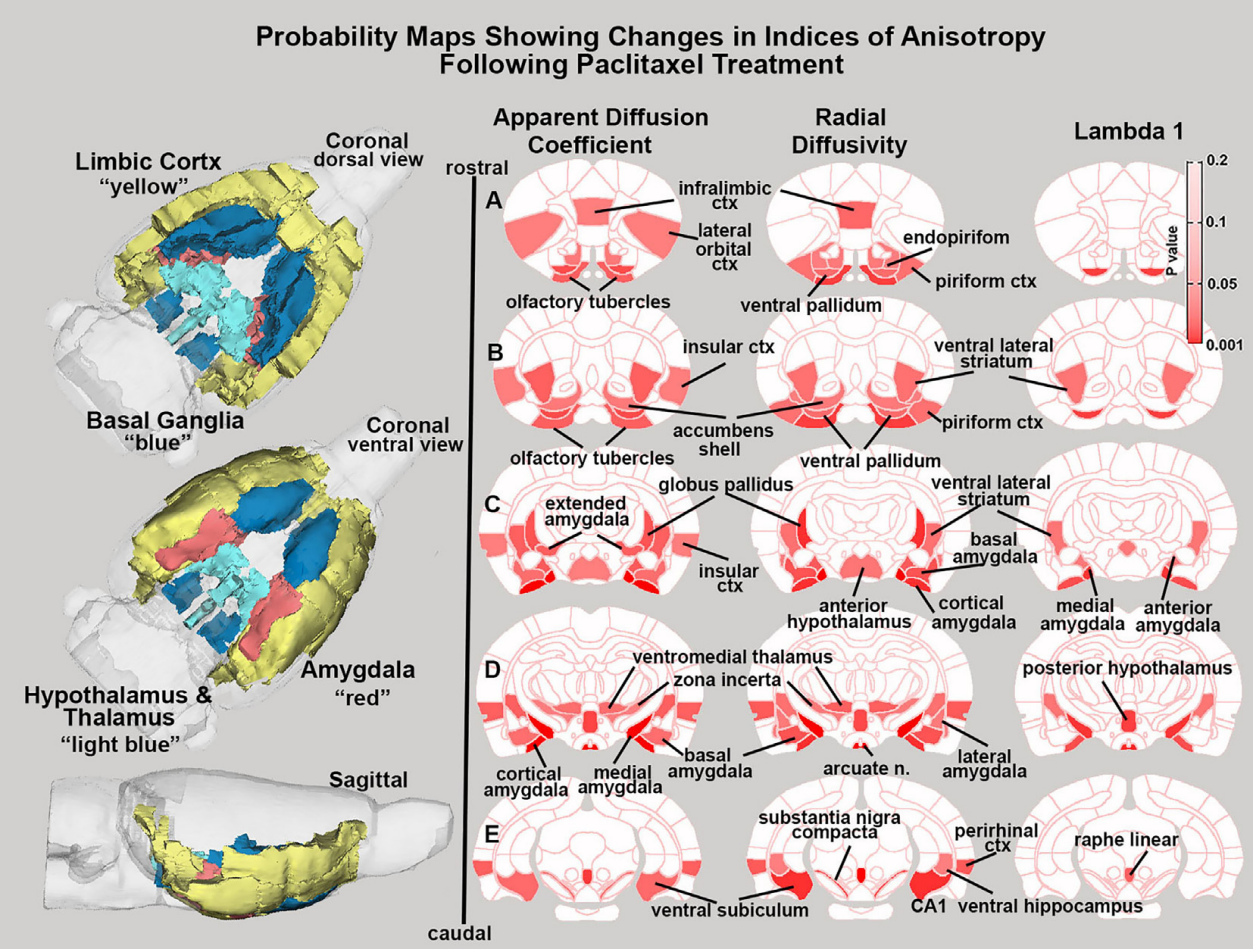
Diffusion Weighted Imaging. The 2D axial images are probability maps showing the location of brain areas with significant changes (pink to red) in the different measures of anisotropy following paclitaxel-treatment. These significant brain areas are clustered into four major brain regions and reconstructed into 3D volumes positioned in the glass brain to the left. (For interpretation of the references to color in this figure legend, the reader is referred to the web version of this article.)

Fig. 2 is A composite of 2D and 3D images showing the changes in indices of anisotropy following paclitaxel treatment as compared to vehicle is shown in Fig. 2. The 2D images are probability maps showing areas (pink to red) significantly affected by paclitaxel for apparent diffusion coefficient, radial diffusivity and lambda 1. Brain areas with input from mesencephalic dopaminergic system are affected (e.g. ventral pallidum, olfactory tubercles, accumbens shell, ventral striatum) as is the limbic cortex (e.g. infralimbic, piriform, insular, perirhinal cortices), amygdala (e.g. basal, cortical, anterior, medial, lateral, and extended amygdaloid areas), hippocampal areas (e.g. CA1, subiculum) and raphe linear. A full list of affected areas for the different indices of anisotropy, ranked in order of their significance from 173 brain areas, can be found in Tables 1S-4S Supplementary Data. These significant brain areas are clustered into four major brain regions and reconstructed into 3D volumes positioned in the glass brain to the left of Fig. 2. These color-coded volumes form a contiguous area extending from the ventral forebrain to the ventral mesencephalon. In all indices of anisotropy, except fractional anisotropy, the paclitaxel-treated rats show increase anisotropy.

### 3.3 Resting state functional connectivity

**Fig. 3:**
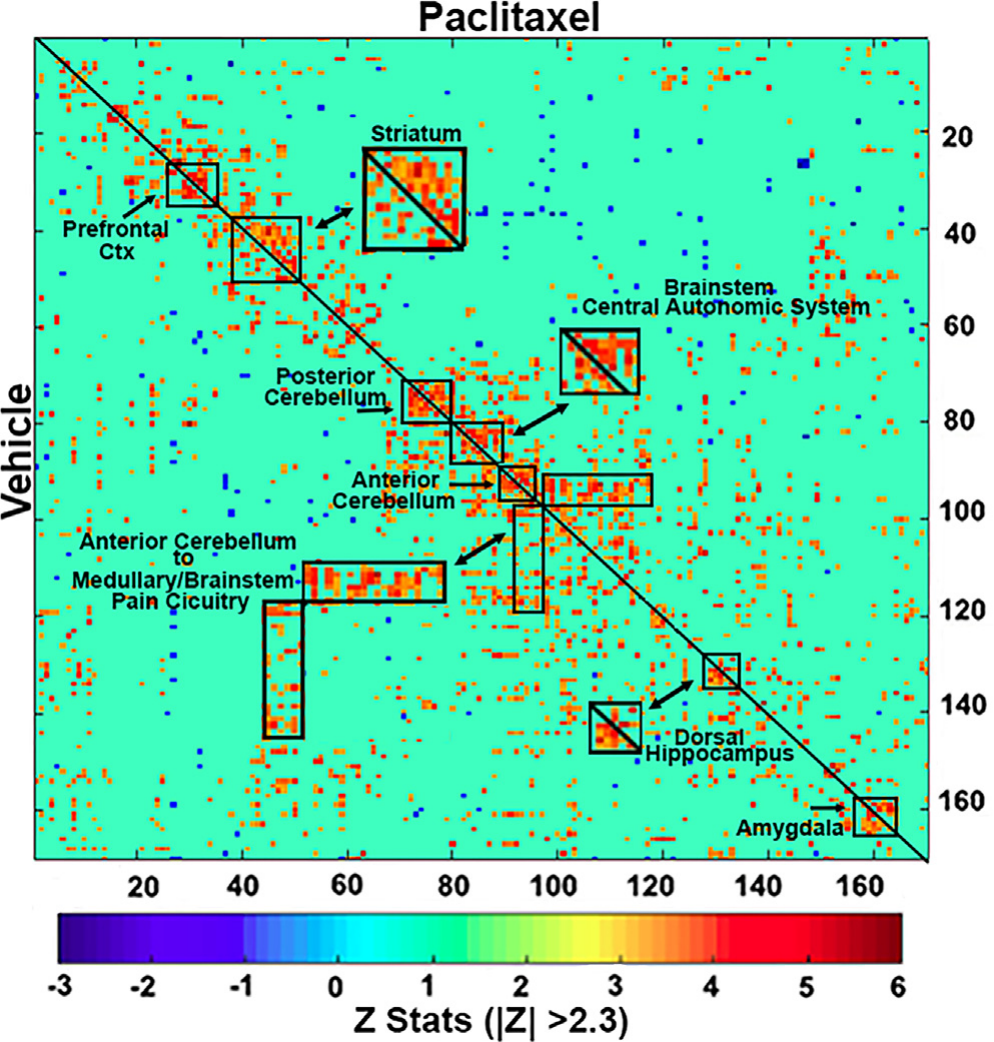
Functional Connectivity Matrix. Shown are correlation matrices of 166 rat brain areas for rsFC comparing vehicle (n = 6) vs paclitaxel (n = 7). Z-scores of Pearson’s correlation coefficients for both groups are displayed in the color-coded matrix. Greater absolute *Z* values indicate greater connections between two region pairs, while smaller absolute *Z* values indicate weaker connections. Significant correlations passing threshold (|Z| > 2.3) are shown in the matrix. Each dark red/yellow pixel represents one of 166 brain areas that is significantly correlated with other brain areas. The delineated brain areas with significant correlations appear as clusters because they are contiguous in their neuroanatomy and/or function. The diagonal line separates the experimental groups. The location of the pixels for each group are a mirror image of each other. Certain brain areas e.g. striatum, are highlighted and magnified showing the difference in connectivity between the experimental conditions. (For interpretation of the references to color in this figure legend, the reader is referred to the web version of this article.)

Correlation matrices between 166 brain areas for rsFC between the vehicle and paclitaxel-treated rats are shown in Fig. 3. The diagonal line separates the two treatments. The pixel locations denote brain areas and are presented as mirror images. The brain areas with significant correlations often appear as clusters because they are contiguous in their neuroanatomy and function. The delineated, annotated areas highlight clusters that are similar or, in many cases, very different between treatments. These primary clusters constitute major brain regions and their significant connections. For example, the striatum shown as a magnified insert clearly shows greater clustering with paclitaxel indicative of hyperconnectivity while cluster representation of the neurocircuitry of the dorsal hippocampus in the magnified insert, shows less clustering with paclitaxel treatment. The insert highlighting connections from the anterior cerebellum to the pain circuitry in the medullary brain stem show increased connectivity with paclitaxel treatment.

**Fig. 4:**
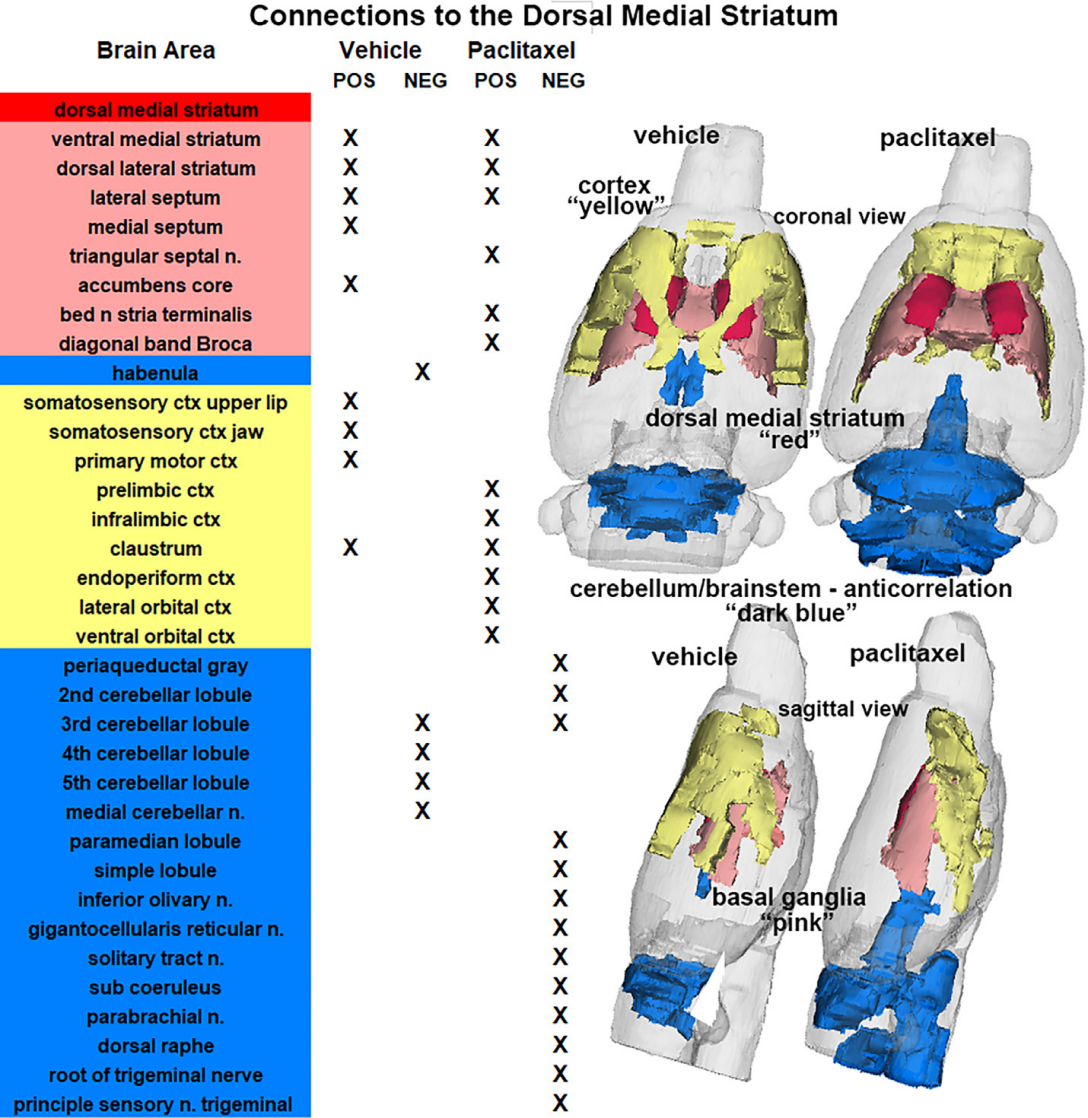
Connections to the Striatum. The significant functional connections following vehicle or paclitaxel treatment to the dorsal medial striatum (highlighted in red) are listed in the table of brain areas that are color coded for their regional organization. With respect to vehicle treatment, the positive connections (Pos) marked by X are to the immediate basal ganglia (pink) and sensory motor cortices (yellow). The negative (Neg) or anticorrelations with vehicle treatment are primarily associated with the cerebellum and habenula (blue). In contrast, paclitaxel treatment shows enhanced positive connectivity in the basal ganglia and limbic cortex and anticorrelation in the cerebellum/brainstem. The 3D organization of these brain areas are shown in the glass brains. (For interpretation of the references to color in this figure legend, the reader is referred to the web version of this article.)

The reorganization of connectivity between the 166 different brain areas for the striatum following paclitaxel treatment is shown in Fig. 4. The color-coded table highlights different brains regions with significant positive and negative connectivity (marked in X) to the dorsal medial striatum (red) from vehicle- and paclitaxel-treated rats. In both experimental groups there is positive connectivity within the striatum (ventral medium and dorsal lateral subareas). The positive connectivity extends to the septum, accumbens core and areas of the sensory motor cortex in vehicle treated rats. The vehicle rats also show negative or anti-correlation between the striatum and cerebellum. In paclitaxel treated rats there is a loss of positive connectivity to the sensory motor cortices that is redirected to the prefrontal limbic cortex (prelimbic, infralimbic, endopiriform, lateral orbital, and ventral orbital cortices). There is also a high level of anti-correlation to cerebellar and brainstem areas with paclitaxel that include several key areas within the pain neural circuit e.g. periaqueductal gray (PAG), gigantocellularis reticular n., solitary tract n. parabrachial n. raphe and sensory n. of trigeminal nerve. The organization of these different brain regions following vehicle and paclitaxel treatment are shown in the 3D brain images. The differences between treatments is the connectivity to the cortex shown in yellow and the anti-correlation to cerebellum and brainstem shown in blue.

**Fig. 5:**
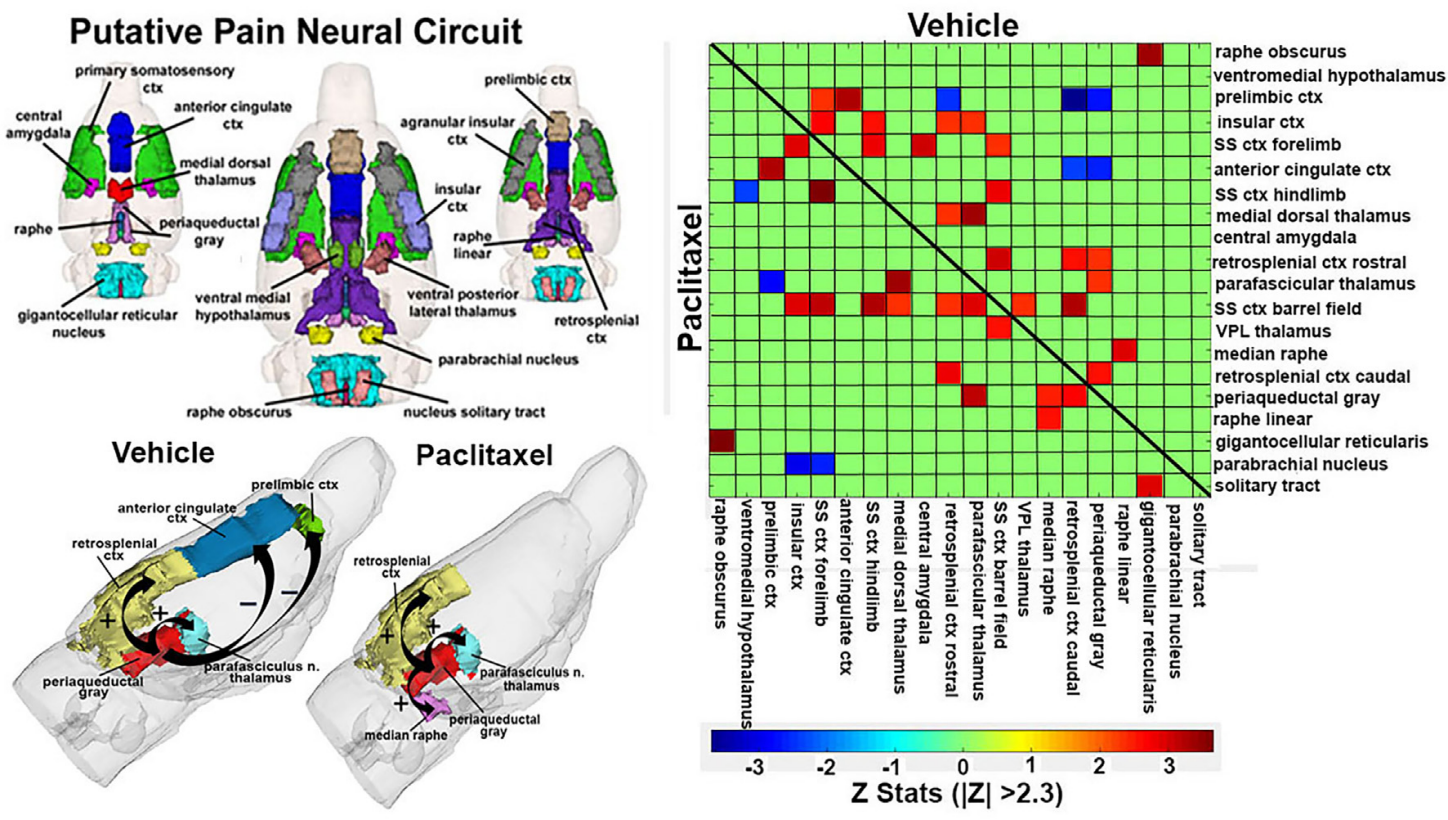
Functional Connectivity Within the Pain Neural Circuit. Shown in the upper left is a 3D representation of the different volumes that comprise the putative pain neural circuit. The central image is a coronal view of a translucent shell of the brain showing the total composite and location of the different 3D volumes. The template used to define the neural circuit of pain in the rat was derived from the work of Gauriau and Bernard (Gauriau and Bernard, 2002) and meta-analyses from various neuroimaging modalities used to study pain in humans (Apkarian et al., 2005). A correlation matrix for each of these 20 brain areas is shown highlighting areas between treatments with a significant positive correlation (red-brown) and a negative or anti-correlation (blue). A 3D schematic proposing the interactions between these brain areas for each treatment is shown to the bottom left. (For interpretation of the references to color in this figure legend, the reader is referred to the web version of this article.)

A reconstruction of the putative pain neurocircuitry is shown in Fig. 5. A correlation matrix for each of these 20 brain areas is shown highlighting areas between treatments with a significant positive correlation (red-brown) and a negative or anti-correlation (blue). A 3D schematic proposing the interactions between these brain areas for each treatment is shown. With vehicle treatment the PAG shows a positive correlation with the parafascicular n. of the thalamus and retrosplenial cortex and anti-correlation with the anterior cingulate and prelimbic cortices. With paclitaxel treatment the anti-correlation is absent and replaced with a positive correlation between the PAG and median raphe.

**Fig. 6:**
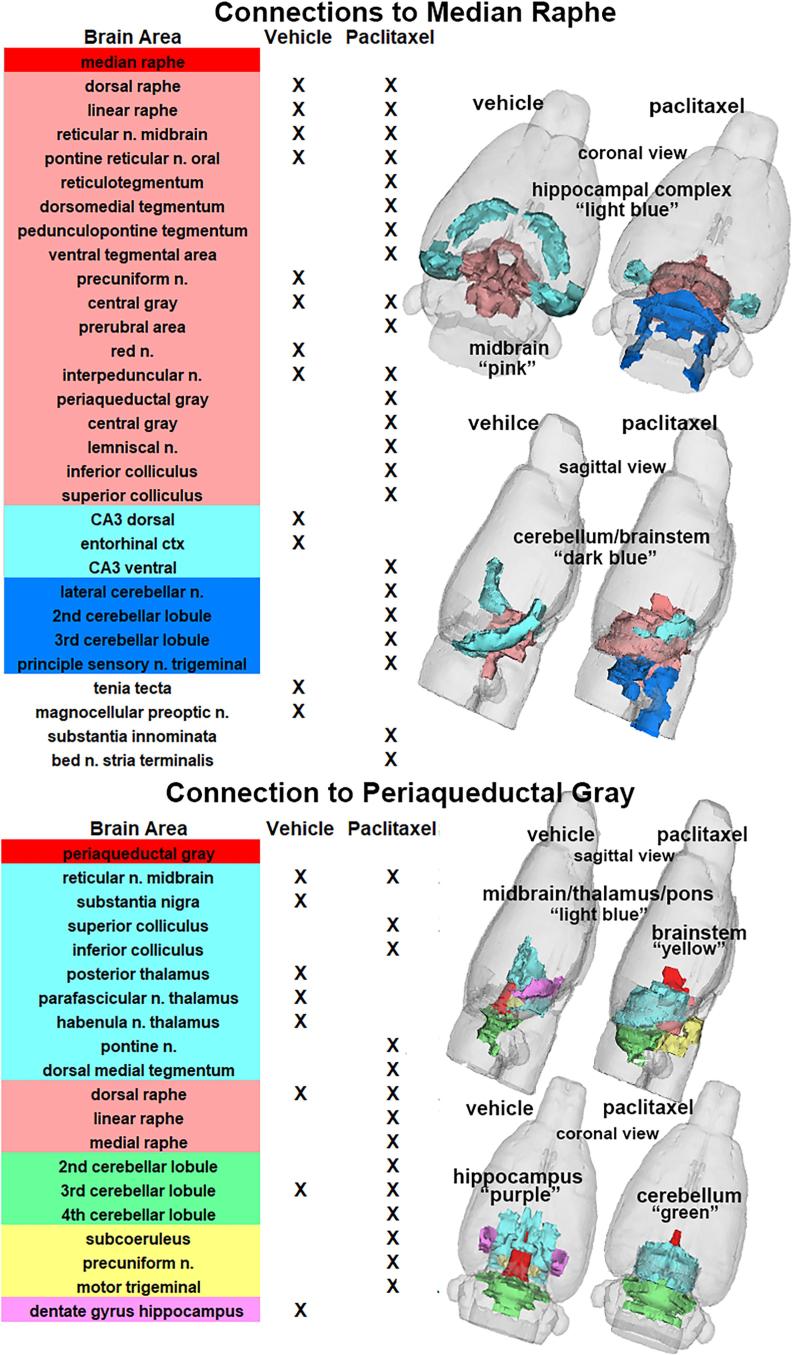
Functional Connections to Raphe and Periaqueductal Gray. The significant functional connections following vehicle or paclitaxel treatment to the median raphe and periaqueductal gray (highlighted in red) are listed in the two tables of brain areas that are color coded for their regional organization as described in the legend for Fig. 4. (For interpretation of the references to color in this figure legend, the reader is referred to the web version of this article.)

The reorganization of connectivity between the 166 different brains for median raphe and periaqueductal gray following paclitaxel treatment is shown in Fig. 6. In the case of the median raphe, there is connectivity within the raphe subregions and with other areas in the midbrain/pons (pink). With paclitaxel treatment there is hyperconnectivity to midbrain/pons that includes several areas of tegmentum, central gray, periaqueductal gray, lemniscus and colliculi. The increased connectivity extends to the cerebellum and sensory n. of the trigeminal nerve. This reorganization is shown in 3D reconstruction highlighting the connectivity of the median raphe to the major brain regions shown in the table. The PAG shows a similar hyperconnectivity to many of the same areas as the median raphe following paclitaxel treatment.

## 4 Discussion

This is a proof of concept study based on numerous reports that paclitaxel in healthy rats can rapidly induced cold allodynia within eight days of treatment (Deng et al., 2012, Panoz-Brown et al., 2017, Rahn et al., 2014, Smith et al., 2017). There are many reports in the human and animal literature that the altered perception of pain from chemo- or injury-induced peripheral neuropathies involves the reorganization of neural circuits in the brain. However, there have been no prospective studies, human or animal, using MRI to identify the changes in brain neural circuitry following treatment with paclitaxel without the confound of cancer. To that end, we used non-invasive diffusion weighted imaging and functional connectivity, to characterize the reorganization of the pain neural circuitry in healthy rats shortly after the cessation of paclitaxel treatment.

### 4.1 Diffusion weighted imaging

While there have been several imaging studies in rodents reporting reorganization of brain structure and function in response to the neuropathic pain caused by spared nerve injury (SNI) surgery (Chang et al., 2014, Baliki et al., 2014, Hubbard et al., 2015, Bilbao et al., 2018, Seminowicz et al., 2009), this is the first that we are aware that uses chemotherapy to induce neuropathic pain and the first to use diffusion imaging to map reorganization. The changes in anisotropy collected from diffusion imaging reveal a neuroanatomical pattern that reflects the involvement of the mesocorticolimbic dopaminergic system e.g. prefrontal cortex, striatum, accumbens, amygdala and hippocampus (Borsook et al., 2007, Apkarian, 2008). These many areas reflect reorganization associated with the emotional-affective component of chronic pain. Quantitative anisotropy reflects the physical properties of proton spins in water that create the contrast that characterize the properties of different tissue. The determinants of indices of diffusion at a microscopic level are many and reflect the gray matter microarchitecture (Kulkarni et al., 2015). The increase in anisotropy noted in these studies could represent a decrease in extracellular water that would be consistent with a decrease in brain volume. Indeed, studies using voxel-based morphometry in rodents following SNI surgery and induction of mechanical allodynia report a decrease in brain volumes in many of these same areas identified with diffusion imaging (Baliki et al., 2011, Bilbao et al., 2018, Seminowicz et al., 2009). Similarly in human subjects, MRI has identified marked reduction of frontal and temporal brain volumes as well as changes in white-matter tract integrity following chemotherapy (Saykin et al., 2003). A reduction in brain volume in these brain areas associated with the mesocorticolimbic dopaminergic system is observed in many types of chronic pain (Baliki et al., 2011, Fritz et al., 2016, Wood, 2010).

### 4.2 Resting state functional connectivity

In these studies, we found changes in functional connectivity in response to paclitaxel as compared to vehicle. When comparing the correlation matrices between vehicle and paclitaxel treatment, the brainstem shows evidence of reorganization with increased connectivity. This is not unexpected given the many studies in rodents and humans showing modulation of chronic pain is mediated by areas in the brainstem (Mills et al., 2018, Youssef et al., 2016, Bouhassira et al., 1993, Ossipov et al., 2014). The striatum showed connectivity within and between the accumbens core, septum and sensory motor cortex. With paclitaxel treatment connectivity within the striatum remained; however, the connections to the sensory motor cortex were lost and replaced with increase connectivity to the prefrontal cortex a key area for reorganization in SNI model thought to be involved in the perception of chronic pain (Metz et al., 2009, Millecamps et al., 2007). This shift from sensory motor cortex to increased connectivity to limbic circuitry (e.g. prefrontal cortex, striatum, PAG, amygdala) occurs within twenty-eight days following SNI surgery (Chang et al., 2014, Baliki et al., 2014). The reorganization of corticostriatal circuitry is hypothesized to be causally involved in the transition from acute to chronic pain (Baliki et al., 2012). The major changes occur in neural circuitry involved in emotion and motivation with the accumbens as a key node (Baliki et al., 2010, Chang et al., 2014, Sagheddu et al., 2015, Becerra and Borsook, 2008). In these studies on chemotherapy-induced neuropathic pain, the accumbens shows connectivity with the dorsal striatum that is not present in paclitaxel-treated rats. This decrease in connectivity between the accumbens and dorsal striatum with neuropathic pain was also reported in the SNI model (Chang et al., 2014). It should be noted, the neuropathic pain following SNI surgery in rats does not involve reorganization in pain neural circuitry (Chang et al., 2014, Baliki et al., 2014).

### 4.3 Periaqueductal gray

Interestingly, the striatum shows anti-correlation with the hindbrain following paclitaxel treatment. This anti-correlation includes key nodes in the pain neural circuit e.g., parabrachial n., gigantocellularis reticular n., raphe, solitary tract n., PAG and principle sensory n. of the trigeminal nerve. This anti-correlation would suggest when the striatum/prefrontal cortex have an increase in functional coupling, the pain neural circuitry in the hindbrain has a decrease in functional coupling. When the putative neural circuitry of pain is isolated from the rest of the brain and examined for functional connectivity within its nodes, the PAG presents as a key area reorganizing its connections following paclitaxel treatment. With vehicle treatment the PAG shows an anti-correlation with prelimbic and anterior cingulate cortices. With reorganization the PAG loses anti-correlation with this part of the limbic cortex, but shows a significant positive correlation with the raphe.

The PAG has a seminal role in the history of pain research as stimulation of this area produces profound analgesia in animals and humans (Reynolds, 1969, Hosobuchi et al., 1977, Richardson and Akil, 1977). The PAG and its projections to the rostral ventral medulla (RVM) (e.g. areas of caudal raphe and gigantocellularis) are part of a descending pain modulatory system. With acute pain, activation of the PAG increases the pain threshold and reduces the perception of pain underscoring importance of the PAG in the descending pain modulatory system (La Cesa et al., 2014). Dysregulation of this system is thought to contribute to neuropathic pain (Vanegas and Schaible, 2004, Heinricher et al., 2009). With chronic pain, the PAG shows increased functional connectivity to the hippocampus, nucleus accumbens, and anterior cingulate cortex adding the elements of emotion and affectation to the modulation of pain (Mills et al., 2018). The modulation of pain from the PAG-RVM to the dorsal root of the spinal cord is preferentially inhibitory; however, this brainstem region has both on and off neurons that are sensitive to inputs from the mesocorticolimbic dopaminergic system to enhance or suppress pain (Heinricher et al., 2009). Deactivation of the PAG-RVM by direct lidocaine microinjection or chemical blockade eliminates the hyperalgesia and allodynia in rats following nerve ligation: however, this deactivation does not pertain to the initiation of neuropathic pain, just its maintenance (Burgess et al., 2002, Wang et al., 2013, Pertovaara et al., 1996).

### 4.4 Onset of neuropathic pain and reorganization

The SNI model presents with mechanical and cold allodynia within five days of surgery; however, reorganization in rats characterized by MRI does not occur until weeks later (Chang et al., 2014, Baliki et al., 2014, Seminowicz et al., 2009). In these studies, cold allodynia was obvious by eight days from the start of paclitaxel treatment. MRI within the following 3–4 days shows evidence of reorganization. A recent study by Bilbao et al in mice report evidence of reorganization as early as 5 days post SNI surgery but these changes are reversed by 12 wks with few differences between sham and SNI mice other than sustained hyperconnectivity to the accumbens (Bilbao et al., 2018). Interestingly, paclitaxel shows limited ability to cross the blood-brain barrier (Cavaletti et al., 2000), but nonetheless produces, profound changes in brain resting state connectivity. These observations are consistent with changes induced by paclitaxel-induced neuropathic pain or other signs of toxicity. The reorganization observed in our studies may be due, in part, to the chemotherapy model where paclitaxel can directly affect the peripheral sensory neurons, including dorsal root ganglia, and via direct and/or indirect actions on the CNS. Paclitaxel can impact the CNS very quickly decreasing cognitive function in humans (Schultz et al., 2003) and rats (Panoz-Brown et al., 2017). Indeed, we previously reported that the present paclitaxel dosing paradigm results in profound reductions in hippocampal neurogenesis and selective deficits in reversal learning in adult rats (Panoz-Brown et al., 2017). By contrast, spatial memory, old learning, new learning, source memory and episodic memory were unaffected by paclitaxel treatment (Panoz-Brown et al., 2017, Smith et al., 2017). The mechanism behind these cognitive deficits may be neuroinflammation as paclitaxel increases levels of inflammatory cytokines, chemokines and activates astrocytes and/or microglia in pain neural pathways (Ahles and Saykin, 2007). We observed modest increases in mRNA expression levels of the astrocyte maker glial fibrillary acidic protein (GFAP) but not the microglial marker CD11b in paclitaxel compared to cremophor-treated rats during the maintenance phase of paclitaxel-induced neuropathic pain (Rahn et al., 2014). Moreover, we also showed that our paclitaxel dosing regimens produce robust increases in mRNA expression levels of the chemokine monocyte chemoattractant protein 1 (MCP1) in lumbar spinal cord (Deng et al., 2015). More work is necessary to determine whether MCP-1 is similarly increased in brain of paclitaxel-treated rats.

### 4.5 Limitations

As an exploratory study, there were several questions left unanswered. 1) These data were collected from a single point in time immediately after confirming the presence of cold allodynia with the presumption that the brain had undergone reorganization of pain neural circuitry. A comparison of behavioral and imaging data collected months later would have shed light on the persistence and recovery from CIPD and the neuroadaptation that occurs with chronic pain. 2) The study was based on a single dose of paclitaxel taken from a well-established model of CIPD (Deng et al., 2012, Panoz-Brown et al., 2017, Rahn et al., 2014, Smith et al., 2017). A study using a range of doses of paclitaxel may have been able to identify a threshold dose of drug that could alter neuronal organization in the absence of cold allodynia providing a better understanding of paclitaxel’s direct effects on the CNS. 3) Immunohistochemical data on neuroinflammation or changes in the extracellular matrix would help to understand the mechanism behind the changes in anisotropy. 4) These studies were limited to males and did not explore gender differences in reorganization of pain neural circuitry. 5) The rsFC data were collected under low dose isoflurane anesthesia to minimize motion artifact and physiological stress (Guilfoyle et al., 2013). Nonetheless numerous studies comparing the anesthetized and conscious states show similar rsFC data (Gorges et al., 2017, Jonckers et al., 2014).

### 4.6 Summary

Within eight days of paclitaxel treatment and induction of neuropathic pain, diffusion weighted imaging identified a number of brain areas e.g. prefrontal cortex, amygdala, hippocampus, hypothalamus and the striatal/accumbens dopaminergic system involved in the emotional and motivations response to chronic pain. This putative reorganization of gray matter microarchitecture forms a continuum of brain areas stretching from the basal medial/lateral forebrain to the midbrain. Resting state FC identified alterations in connectivity in the brainstem, particularly between nodes in the pain neural circuitry. Unlike previous studies in rat neuropathic pain models, paclitaxel treatment affects reorganization of pain neural circuitry, particularly the connections to the PAG. These data using different imaging modalities fit the notion that chronic pain is regulated by emotion and motivation that influence activity in the PAG and brainstem to modulate pain perception. In theory, MRI could be used in the clinic to titer the dose and duration of chemotherapy based on the disruption of anticorrelation between the prefrontal ctx/striatum and the PAG/brainstem
